# The Good and the Bad of Natural Killer Cells in Virus Control: Perspective for Anti-HBV Therapy

**DOI:** 10.3390/ijms20205080

**Published:** 2019-10-13

**Authors:** Paola Fisicaro, Marzia Rossi, Andrea Vecchi, Greta Acerbi, Valeria Barili, Diletta Laccabue, Ilaria Montali, Alessandra Zecca, Amalia Penna, Gabriele Missale, Carlo Ferrari, Carolina Boni

**Affiliations:** 1 Laboratory of Viral Immunopathology, Unit of Infectious Diseases and Hepatology, Azienda-Ospedaliero-Universitaria di Parma, 43126 Parma, Italy; pfisicaro@ao.pr.it (P.F.); rossi.marzia@gmail.com (M.R.); 2007arandy@gmail.com (A.V.); greta.acerbi@gmail.com (G.A.); barili.valeria@gmail.com (V.B.); dilettal@hotmail.com (D.L.); ilaria.montali1@studenti.unipr.it (I.M.); alessandra.zecca@studenti.unipr.it (A.Z.); apenna@ao.pr.it (A.P.); missalegabriele@gmail.com (G.M.); cferrari00@gmail.com (C.F.); 2 Department of Medicine and Surgery, University of Parma, 43126 Parma, Italy

**Keywords:** chronic HBV infection, NK cell, immune-therapy, NK-T cell interplay

## Abstract

Immune modulatory therapies are widely believed to represent potential therapeutic strategies for chronic hepatitis B infection (CHB). Among the cellular targets for immune interventions, Natural Killer (NK) cells represent possible candidates because they have a key role in anti-viral control by producing cytokines and by exerting cytotoxic functions against virus-infected cells. However, in patients with chronic hepatitis B, NK cells have been described to be more pathogenic than protective with preserved cytolytic activity but with a poor capacity to produce anti-viral cytokines. In addition, NK cells can exert a regulatory activity and possibly suppress adaptive immune responses in the setting of persistent viral infections. Consequently, a potential drawback of NK-cell targeted modulatory interventions is that they can potentiate the suppressive NK cell effect on virus-specific T cells, which further causes impairment of exhausted anti-viral T cell functions. Thus, clinically useful NK-cell modulatory strategies should be not only suited to improve positive anti-viral NK cell functions but also to abrogate T cell suppression by NK cell-mediated T cell killing. This review outlines the main NK cell features with a particular focus on CHB infection. It describes different mechanisms involved in NK-T cell interplay as well as how NK cells can have positive anti-viral effector functions and negative suppressive effects on T cells activity. This review discusses how modulation of their balance can have potential therapeutic implications.

## 1. Introduction

About 250 milion people worldwide are chronically infected with hepatitis B virus (HBV). Therefore, HBV represents a major healthcare problem [1]. Treatment for patients with chronic hepatitis B (CHB) is limited to a few antiviral drugs that suppress viral production without eradicating HBV from the liver [2,3]. Since the immune response is recognized as functionally defective in patients with chronic HBV infection [4,5], it is widely accepted that these patients would benefit from strategies aimed at an immune functional reconstitution, either alone or in combination, in order to complement the effect of current antiviral therapies. Among the potential cellular targets of immune modulatory therapies for chronic hepatitis B infection, Natural Killer (NK) lymphocytes represent possible candidates, due to their key role in anti-viral control.

NK cells have been considered as part of the innate arm of the immune response, which belong to the family of innate lymphoid cells (ILC), since they do not express receptors encoded by rearranging genes [6]. They were initially identified in the mouse for their rapidly induced cytolytic activity against tumor cells in the absence of antigen immunization [7,8,9,10]. It is now well known that NK cells can be cytotoxic to virus-infected cells or cancer cells, both in the absence of antibodies or through antibody-dependent cell cytotoxicity (ADCC), due to the constitutive expression of perforin and granzymes, or to the interaction with target cell death receptors. Moreover, they are recognized as major producers of cytokines and chemokines [11].

Lacking antigen-specific receptors, NK cell activation depends on the integration of inhibitory and activating signals from cell surface receptors. Attacking healthy cells is prevented thanks to the recognition of HLA-class I molecules by specific inhibitory receptors [12], which are represented by members of the killer immunogobulin-like receptors KIR/CD158 family, LIR-1, and the CD94/NKG2A heterodimer. The latter is typical of an earlier NK cell maturation stage than KIRs, with KIRs-NKG2A co-expression detectable only on a subset of maturing cells [12,13]. Target cells may become susceptible to NK killing by down-regulating MHC class I molecules when stressed, i.e., viral infection or neoplastic transformation, which is a phenomenon called “missing-self” [11]. However, stressed, transformed, or infected cells can also upregulate different molecules that trigger activating NK cell receptors. These are mainly represented by the “natural cytotoxicity receptors” (NCR), specifically NKp46, NKp44, NKp30, and NKG2D, even though activating forms of KIRs have been described [12]. Moreover, several studies reported that toll-like receptor (TLR) expression can mediate a strong NK cell activation [14]. Down-regulation of activating receptors and up-regulation of inhibitory receptors have been reported as the prevalent NK cell phenotypic signature in tumors and chronic infections, which results in impaired effector functions that may contribute to viral persistence and tumor growth [15].

## 2. NK Cell Subsets

Two main NK cell subpopulations can be identified in peripheral blood by the expression levels of the adhesion molecule CD56 and the low affinity Fc receptor CD16 (FcγRIII): a smaller CD56^bright^ CD16^neg^ subset (CD56^bright^), which can produce high amounts of cytokines but shows low expression of lytic granules, and a larger CD56^dim^ CD16^bright^ NK subset (CD56^dim^), which is more cytolytic with lower capacity to produce cytokines [13] (Table 1). It is generally accepted that CD56^bright^ cells represent the precursors of the more mature, terminally differentiated CD56^dim^ NK cells. The two subsets differ in homing properties, surface expression of HLA-I-specific receptors, and metabolic features. CD56^bright^ NK cells preferentially migrate to secondary lymphoid organs, express only CD94/NKG2A, up-regulate metabolism more efficiently in response to cytokines, express higher levels of the glucose transporter Glut1 with more efficient glucose uptake, and are more glycolytic. CD56^dim^ NK cells preferentially migrate to inflamed peripheral tissues, express KIRs and/or LIR-1, are heterogeneous in their metabolic response to cytokines, and have low basal expression of Glut1 [12,16].

NK cell subsets also display a different pattern of inhibitory checkpoints. High surface PD-1 expression, mostly confined to CD56^dim^ NK cells, is detectable in about 25% of healthy donors, and in a higher frequency of cancer patients where it has been associated with a reduced NK cell functionality. PD-1+ NK cells display low cytolytic activity and altered capability of releasing IFN-γ and TNF-α cytokines after stimulating with tumor targets [26].

PD-1 expression has been detected as a cytoplasmic pool of PD-1 mRNA transcripts and proteins in all NK cell subsets, although higher in CD56^dim^ than in CD56^bright^ cells. This has also been observed in surface PD-1 negative resting NK cells from healthy subjects, as well as in tumor-associated NK cells, which is consistent with the possibility of a prompt surface expression in response to the appropriate stimuli [27]. A novel checkpoint inhibitor specifically associated with NK cell maturation and effector functions is the interleukin-1 receptor 8 (IL-1R8), which has been found to be overexpressed in CD56^dim^ NK cells, as compared to the CD56^bright^ subset as well as T and B lymphocytes [28]. Other important regulators of NK cell functions include adhesion molecules that bind nectins and nectin-like family proteins, such as TIGIT, CD96, and CD226 (DNAM-1). CD96 and TIGIT, can bind to CD226 ligands and counterbalance CD226-mediated NK cell activation [26,29]. More recently, NKG2A has been described in a mouse model of chronic HCV infection as an important checkpoint implicated in the impaired function of hepatic NK cells [30]. Moreover, mice deleted for the deubiquitinase Otub1 showed potentiated anti-cancer NK and CD8 cell responses, which allows us to identify Otub1 as a crucial regulator of NK and CD8 cell homeostasis, due to its role in IL-15R signaling transduction [31].

In addition to CD56^bright^ and CD56^dim^ cells, a subset of CD56^neg^ CD16^bright^ NK cells has been identified as a minor subpopulation in peripheral blood from healthy subjects despite being significantly expanded in patients with chronic HIV and HCV infections [19] as well as in CMV/EBV co-infected older, healthy donors [20]. A recent proteome analysis of CD56-negative NK cells has allowed the authors not only to recognize several shared features with CD56^dim^ cells but also some differences in surface receptor expression, e.g., a lower cytotoxic capacity, despite high expression of perforin and granzymes H and M, and a reduced IFN-γ production upon stimulation [32]. Unconventional CD56^dim^ CD16^neg^ NK cells have also been observed in patients with leukemic malignancies, present at significantly higher frequency as compared to healthy donors. In physiological conditions, these lymphocytes are considered multi-functional, which displays both high cytotoxicity and IFN-γ production potential, even though they resulted in not being fully functional when associated with malignancies [17,18] (Table 1).

## 3. Liver NK Cells

There is ample evidence that tissue resident NK cells differ phenotypically and functionally from NK cells in lymphoid organs or in circulation. While representing only a small fraction, ranging from 5% to 15% of peripheral lymphocytes, NK cells account for up to 50% of intrahepatic lymphocytes, where, besides having a role in immune surveillance, they have been reported to also play an anti-fibrotic activity through IFN-γ production or by killing activated stellate cells (HSC), responsible for collagen production in response to hepatocyte damage [33]. Hepatic NK cells were demonstrated to be more cytotoxic than their peripheral counterparts upon in vitro cytokine stimulation [34]. A recent study associates the lethal liver failure that occurred in a case of fulminant hepatitis A to the excessively elevated levels of serum and hepatic IL-18 due to the genetic deficiency for the IL-18 binding protein gene (IL-18BP). This gene encodes an IL-18 neutralizing/buffering protein. Uncontrolled IL-18 production likely stimulated an exasperated NK cytotoxic activity [35], since IL-18, together with other γ-chain cytokines, can significantly intensify NK effector functions [36].

Hepatic NK cells have been distinguished into conventional (cNK) and liver resident NK cells (lrNK) (Table 1). The latter are predominantly CD56^bright^ CD16^low^, CD69 positive, T-bet^lo^Eomes^hi^, express low levels of perforin and granzyme B, and are less cytotoxic and less able to produce pro-inflammatory cytokines than circulating NK cells [23]. Moreover, they are enriched in the chemokine receptors CCR5 and CXCR6 that are poorly expressed on peripheral NK cells and can mediate liver homing by interacting with their ligands, CCL3, CCL5, and CXCL16, which are highly expressed within liver sinusoids on Kuppfer cells, T, and NK lymphocytes and liver sinusoidal endothelial cells (LSECs) [22,37]. It has been proposed that, upon recruitment from circulation, Eomes^lo^CXCR6+ NK cells rapidly up-regulate Eomes, as well as chemokine receptors and integrins that mediate intrahepatic retention. Therefore, this allows their persistence within the liver for many years [38].

Liver resident CD56^bright^ CD16^low^ cells display a different gene expression and phenotypical profile as compared to their circulating counterparts, while hepatic CD56^dim^ CCR5^neg^CXCR6^neg^ NK cells resulted in transcriptionally similar peripheral CD56^dim^ NK cells, which indicates that they are likely able to circulate through the liver without being retained [22]. Moreover, experiments in mice have recently shown that lrNK and cNK cells exert distinct functions, with the latter promoting the T cell function, while lrNK display a regulatory role on anti-viral T cell responses during acute and chronic viral infections through the PD-1/PD-L1 signaling, which contributes to liver tolerance [21].

Remarkably, CXCR6+ lrNK cells can acquire memory to haptens and viral antigens. Long-lived “memory-like” NK cell subsets, which are CD226-dependent for their maturation, have recently been described. They can differentiate after exposure to pathogens, which provides enhanced effector responses upon the secondary antigen challenge, and can express enhanced ADCC and IFN-γ production and display transcriptional and epigenetic remodeling [24,25] (Table 1).

## 4. NK Cells in HBV Infection

While HBV was reported to behave as a ‘‘stealth’’ virus, which is poorly seen by innate immunity receptors, and is poorly able to induce innate responses during the first weeks of infection in chimpanzees [39], in humans, a number of studies described NK cell activation following acute HBV infection. This is shown by raised peripheral NK cell frequencies preceding the ALT peak [40] and increased expression of activation markers [41,42,43,44]. In addition, the NK cell function enhanced during acute HBV infection [41,43] while temporarily inhibiting by interleukin-10 production in correspondence with the serum HBV-DNA peak, as detected in the early preclinical phase of acute HBV infection [45]. A more recent study showed that peripheral NK cells from acutely HBV-infected patients are selectively perturbed within the CD56^dim^ subset, which identifies specific phenotypic profiles strongly associated with, and predictive of, an early or late HBsAg clearance, on the basis of CD94, NKp30, and CD161, or on KIR3DL1, CD158a, NKp46, and perforin expression, respectively. Functional analysis revealed that elevated antibody-mediated cytotoxicity degranulation can represent a key mechanism in early clearance. On the other hand, higher cytokine production was detected in the case of later clearance, which suggests that ADCC-mediated NK cell killing is more effective than non-cytopathic mechanisms in the early control of acute HBV infection [46].

In chronic HBV infection, NK cell phenotype and function may vary in relation to the infection stage. Some conflicting results have been reported due to the wide spectrum of different clinical conditions characterizing the natural history of HBV infection [3]. As compared with healthy donors, a high expression of some activation/proliferation markers and death ligands (TRAIL) was observed both on peripheral and intrahepatic NK cells from chronic patients [47,48,49], particularly in the active hepatitis stage [50,51,52,53,54,55,56]. Intrahepatic NKG2D up-regulation was proposed to mediate NK activation and liver inflammation, which was particularly amplified in patients with acute-on-chronic liver failure (ACLF) [57]. However, such an activated phenotype is not associated with a significant potentiation of the NK cell function [58,59]. The dichotomy of a decreased cytokine production in the presence of a conserved or improved cytotoxicity, which likely accelerates liver damage, has been described in treatment-naive patients [47,48,50,51,53,54,56]. Recent reports associate NK cell dysfunction to a downregulation of the IL-2/IL-15 receptor β chain CD122 [60], or to the reduced expression of STAT3, which exerts a positive regulatory effect on NKp46 transcription [61].

**Figure 1:**
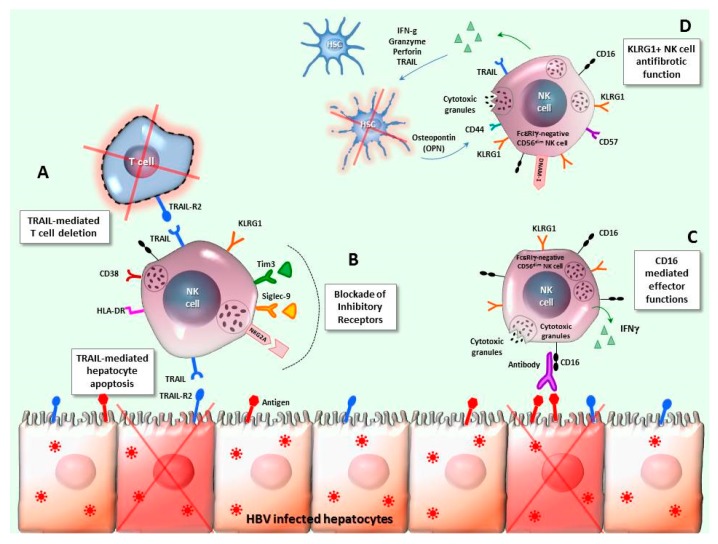
NK cell features in chronic HBV infection. A: The interaction between TRAIL, which is up-regulated on intrahepatic NK cells, and its receptors represents a mechanism for NK cell-mediated lysis of HBV-infected hepatocytes, but also a pathway leading to the deletion of HBV-specific T cells overexpressing the TRAIL-R2 receptor. B: The NK cell dysfunction observed in CHB infection has been targeted by correction interventions based on up-regulated inhibitory receptors blockade, like Tim-3, NKG2A, and Siglec-9. C: FcεRIγ-negative memory-like CD56^dim^ NK cells have been detected in patients with chronic HBV infection, particularly in those co-infected with HCMV, as compared with healthy donors. This NK cell subset shows increased CD16-mediated effector functions and a distinct metabolic and epigenetic signature. D: KLRG1+ NK cells displaying a mature phenotype, with elevated CD57 and DNAM-1 expression, have been found enriched in CHB patients, within the FcεRIγ-negative memory-like CD56^dim^ NK cell subpopulation. Such cells can be stimulated by osteopontin (OPN), produced by activated hepatic stellate cells (HSC), and exert an anti-fibrotic effect by killing HSC in a TRAIL-dependent manner.

During long-term nucleos(t)ide (NUC) analogue therapy, no significant improvement of the NK cell function was observed, despite a reduced expression of some activation/proliferation markers, including the death ligand TRAIL that indicates the attenuation of the NK pro-inflammatory phenotype (Figure 1) [48,50]. However, a role for NK cells in HBsAg seroclearance was suggested by the increase in both degranulation and cytokine production, along with CD38 upregulation, in correspondence of the ALT flares following NUC discontinuation, which was particularly enhanced in the fraction of patients experiencing HBsAg loss [62].

Moreover, a significantly increased CD56^bright^ NK cell expansion, activation, and IFN-γ production was observed throughout PegIFNα therapy [63,64,65], and then maintained by the sequential administration of NUC [66].

Different studies have been performed to improve the NK cell function in chronic HBV infection, by blockade of inhibitory receptors, including Tim-3, NKG2A, Siglec-9 [67,68,69] (Figure 1), or of the immune-suppressive cytokines IL-10 and TGF-β. An influence of the immune-suppressive hepatic environment was documented in inducing the NK cell alteration typically detected in chronically-infected patients [50,70] and suppressive cytokines play an important role. In line with this, in vitro experiments showed that uptake of HBV+ exosomes isolated from CHB patient sera by NK cells caused their functional depression, which was further amplified in the presence of TGF-β [71].

Lastly, memory-like polyfunctional NK cells have been detected in CHB patients coinfected with HCMV [72,73]. These long-lived adaptive NK cell responses characterized by the upregulation of NKG2C and the lack of the adaptor protein FcεRIγ, have been widely described in CMV-infected mice and humans [74,75]. Memory-like FcεRIγ-negative CD56^dim^ NK cells have recently been identified as significantly expanded in chronic HBV-HCMV co-infection. Compared with conventional FcεRIγ+CD56^dim^ NK cells, this NK subpopulation displays different metabolic properties, including a higher fraction of functional, polarized mitochondria, and a stable epigenetic signature, along with increased CD16-mediated degranulation potential, which skews the whole NK cell population toward a higher CD16 sensitivity with enhanced ADCC. These data show the mutual influence of HBV- and HCMV-persisting infections on the NK cell repertoire, which significantly affects the immune response to chronic HBV infection, and point to the usefulness of HCMV co-infection evaluation in the application of immunotherapeutic approaches targeting NK cells for an HBV cure [49]. Moreover, KLRG1 upregulation has been detected on circulating and intrahepatic memory-like FcεRIγ-CD56^dim^ NK cells from CHB patients, which characterizes a subset with anti-fibrotic function exerted by a strong TRAIL-mediated induction of hepatic stellate cell (HSC) apoptosis. Thus, KLRG1 expression identifies a beneficial subpopulation of NK cells that is enriched in patients with no liver fibrosis or mild liver fibrosis. It is inversely correlated with serum markers of liver injury, and activated by HSC osteopontin (OPN) thanks to the high expression of the OPN receptor CD44 [76] (Figure 1).

## 5. NK/T Cell Interplay

NK cells not only contribute to the early innate immune response but also act as modulators of T cell responses through indirect mechanisms such as by regulating APC activity or modulating antigen availability and also directly enhancing or suppressing T cells, by releasing or consuming cytokines or by killing T cells [77,78,79,80,81].

### 5.1. Indirect Mechanisms

**Figure 2:**
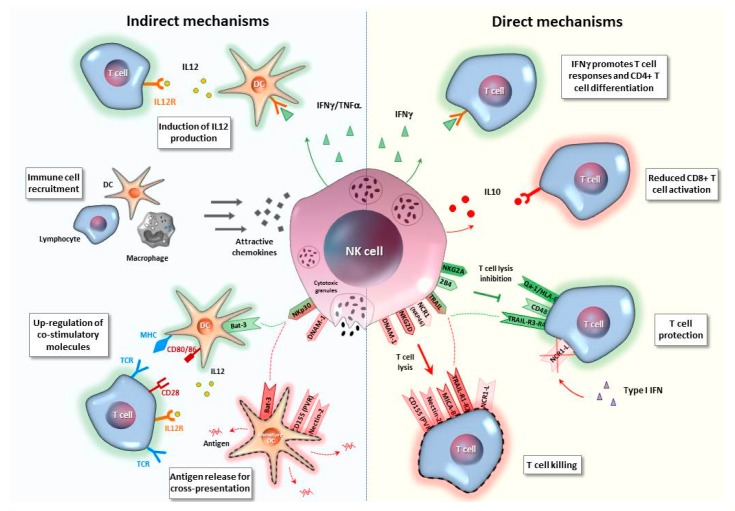
NK/T cell interplay. NK cells can exert either a regulatory or a protective role on T cells via indirect or direct mechanisms. Among indirect interactions, NK cells can influence T cells by regulating dendritic cells (DC), which are responsible for antigen presentation and subsequent T-cell activation. IFN-γ produced by NK cells enhances DC maturation, recruitment, and secretion of IL-12, which, in turn, stimulates T-cell responses. Moreover, NK cells are responsible for the migration of different immune cells through chemokine production. Interaction between NK receptors and their ligands on DC can induce an enhanced antigen presentation capacity, by upregulating DC MHC and costimulatory molecule expression, but can also lead to immature DC lysis, with an antigen release for cross-presentation by DC subsets. NK cells can also directly promote or restrain T-cell responses through IFN-γ or IL-10 release, respectively. Depending on the balance expressed by the different receptor/ligand pairs, NK-T cell cross-talk can result in inhibition or induction of T-cell lysis.

Among indirect T cell modulatory pathways (Figure 2 and Table 2), NK cells can target dendritic cells (DCs) with positive or detrimental effects for T cell responses. NK cells can enhance DC maturation, induces up-regulation of costimulatory molecules and IL-12 production [77,82,83,84]*,* and leads to an increased capacity of DCs to stimulate adaptive T cell immunity. Moreover, NK cells have been reported to favor DC and T-cell recruitment to lymph nodes during influenza infection in mice [85], and, more recently, to stimulate DC migration to the tumor microenviroment, which promotes cancer immune control [86,87]. Furthermore, NK-cell mediated killing of target cells can also promote cross presentation of antigens by DCs that lead to Ag-specific CD8 T-cell activation [88]. This functional role of NK cells as key modulators of multiple DC functions leads to antigen cross-presentation. Stimulation of adaptive immune responses has also been well-highlighted in the setting of tumor surveillance [89,90].

However, NK cells can also negatively regulate T cell immunity by reducing antigen presentation and APC capacity [79,111]. Specifically, they can directly recognize and kill DCs [92,93,94], and can reduce the stimulatory capacity of DCs, which is described in a mouse model of chronic LCMV infection by NK depletion experiments [91]. Lastly, NK cells can modulate antigen availability by regulating the amount of antigen levels [95].

Moreover, a reduced pDC function leading to the disruption of pDC/NK cells interplay has been associated with viral persistence in patients with a CHB infection [112,113].

### 5.2. Direct Mechanisms

Regulation of adaptive immunity by NK cells is also directly sustained by cytokine secretion or by their capacity to recognize and kill activated T cells through cytolytic granules, or death receptor pathways, as well as by the NK cell capacity to process and present antigens to T cells. This was recently described for a subset of effector-memory CD4 T cells [114] (Figure 2 and Table 2).

#### 5.2.1. Cytokine-Mediated Interaction

NK cell effector functions include the production of a variety of cytokines provided with antiviral activity, i.e., IFN-γ, promoting CD4 T cell differentiation, and enhancing T cell responses. In addition, NK cells produce other pro-inflammatory cytokines (TNF-α, chemokines, and GM-CSF) that can regulate anti-viral immune responses [96]. Recently, distinct NK-cell subsets, based on the expression of receptors binding self-MHC-I molecules, have shown different functional capacities in migratory, effector, and immunoregulatory functions on a dendritic cell and antigen (Ag)-specific CD8+ T cell responses during influenza and murine cytomegalovirus infections [115]. However, impairment of T-cell responses can occur via IL-10-dependent and TGF-β-dependent immunosuppressive mechanisms of NK cells that are strongly involved in limiting the antiviral T-cell function [79].

#### 5.2.2. Receptor/Ligand NK-T Cell Cross-Talk

The finding that T cells are susceptible to NK-cell-mediated killing was first demonstrated in murine models of persistent viral infections, which shows a vital rheostat function for NK cells in regulating T-cell responses [80,81].

NKG2D-dependent killing of T cells has been demonstrated in vitro [101,102,116] and in murine models in vivo [81]. NKG2D ligands belong to the MIC (MICA-B) and ULBP (ULBP1-6) families, [117] and are usually not expressed by resting T cells, even though their up-regulation can be triggered by different stimuli, including infections. The final consequence is the direct elimination of activated T lymphocytes by NK cells via NKG2D/NKG2DL-dependent mechanisms, as described in LCMV-infected mice [81]. Moreover, regulation of T-cell responses by a direct perforin-dependent NK-cell-mediated elimination of CD4 T cells, which leads to the loss of help for CD8 T cells, was reported in the same murine model of chronic viral infection [80]. The DNAM-1/PVR axis also plays a role in the NK cell-mediated lysis of activated T cells, which represents a further mechanism taking part in the negative modulation of T-cell responses, through PVR upregulation on activated T cells [103]. Conversely, low expression of ligands for NKG2D and DNAM-1 receptors contributes to render tumor cells more resistant to NK cell-mediated recognition and killing [118,119].

In addition, TRAIL is an NK-cell receptor involved in direct recognition and subsequent death of T cells. Apoptosis of HBV-specific CD8 T cells with upregulated death-inducing receptor TRAIL-R2 can be caused by TRAIL-positive NK cells in patients with CHB infection, as supported by the observation that TRAIL blockade in vitro can enhance HBV-specific CD8 T cell responses [105]. The NK cell mediated regulation of T cell immunitywas also demonstrated during chronic murine cytomegalovirus (MCMV) infection where NK cells specifically eliminated activated CD4+ T cells in the salivary gland by a TRAIL-dependent mechanism [104]. Since T-cell killing is NKG2D-dependent and TRAIL-dependent, naïve T cells are generally protected from killing, while activated T cells become susceptible through the up-regulation of NKG2D and TRAIL ligands [81,101,102,105,116].

A critical role for the NK cell activating receptor NCR1 (NKp46) in the negative regulation of T cell immunity was reported in an LCMV infection [106]. Notably, type I IFNs suppress the expression of the NCR1 ligand on T lymphocytes, which protects T cells against the NK attack mediated by the activating receptor NCR1. As a result of this mechanism, T cells lacking the receptor for type I IFN (IFNAR) were directly killed by NK cells via NCR1 [106]. Recently, the NCR1 effect on T-cell responses has been further investigated during acute and chronic LCMV infection by using an NCR1-deficient (NCR1^gfp/gfp^) mouse model. The absence of NCR1 increased the numbers of virus-specific CD8 T cells that leads to enhanced virus control during acute and chronic LCMV infection. However, the increased CD8 T cell responses caused a concomitant pronounced immunopathology in the setting of chronic infection [107]. Furthermore, transfer experiments of virus-specific CD8 T cells into NCR1-deficient mice revealed a direct NK-T cell cross-talk with killing of activated CD8 T cells in an NCR1-dependent manner, which shows a new pathway engaged by NK cells to modulate adaptive T-cell immunity to protect the host from severe immune-mediated damage [107].

Recent studies indicate that NCR1 is able to control TRAIL expression in NK cells and in innate lymphoid cells (ILCs), which reveals an unexpected link between NKp46 and TRAIL. ILCs from NKp46-deficient mice failed to express normal levels of TRAIL on the surface, which ultimately produced a reduced cytotoxicity toward TRAIL-receptor positive T cells [120,121].

Thus, all these findings strengthen the role of the NK-cell population as a rheostat modulator of anti-viral T cells and suggest that blocking NCR1 on NK cells might represent a possible method to stimulate T-cell responses in chronic viral infections.

Other mechanisms were reported to intervene in NK/T cell calibration operating during chronic viral infections. The interplay between the inhibitory NK-cell receptor 2B4 that belongs to signaling lymphocytic activation molecule (SLAM) family receptors (SFRs), and the CD48 ligand expressed on T cells, was described as playing an important role because it has been implicated in the protection of activated T cells during LCMV infection. Removal of 2B4 from NK cells or CD48 from T cells resulted in increased lysis of activated CD8 T cells by NK cell-mediated killing [98]. More recently, and in line with this evidence, SLAM-family-receptor deficient mice displayed enhanced NK cell activation in response to activated target T cells, which led to defective anti-viral T cell immunity, mainly through effects mediated by 2B4 [99].

#### 5.2.3. Checkpoint Inhibitory Pathways

Moreover, checkpoint inhibitory pathways were described in both T and NK cells. Besides the PD-1/PD-L1 signaling [122], NKG2A emerged as a further potential inhibitory checkpoint expressed both on T and NK cells [108]. Half of the peripheral blood NK cells express NKG2A that can be upregulated when stimulated with cytokines. For CD8 T cells, NKG2A is present in about 5% of the peripheral population at the steady state, but it is further induced by chronic antigen stimulation. Binding of NKG2A/CD94 to its cognate ligand, which is represented by non-classical MHC class I molecules (HLA-E in humans or Qa-1b in mice), inhibits T-cell and NK-cell effector functions.

In LCMV-infected mice, the interaction between the inhibitory murine ligand Qa-1b on T and B cells and the NK cell receptor NKG2A was reported to suppress regulatory NK cell activity and promote anti-viral T-cell immunity. Absence of Qa-1b resulted in enhanced NK cell-mediated regulation of adaptive T-cell immunity in LCMV, which led to more viral replication [100]. By this mechanism, lymphocytes negatively modulate NK-cell function during viral infections in order to increase T-cell responses.

Furthermore, NKG2A was shown to contribute to the inhibition of HIV-infected target cell clearance by NK cells, which suggests that the therapeutic blockade of NKG2A/HLA-E interaction may be beneficial in patients with HIV [109]. Blocking the inhibitory NKG2A receptor can also enhance tumor immunity by promoting both NK and CD8 T cell effector functions in mice and humans [108].

In addition, NKG2A is often co-expressed with PD-1 on activated CD8 T cells [108]. Since PD-1 expression is a hallmark of exhaustion for both CD8 and NK cells [15,24,122], the use of a combination of mAbs blocking the PD-1/PD-L1 and NKG2A/HLA-E inhibitory pathways represents a rational strategy to rescue T- and NK-cell functions. A clinical trial based on such a combination approach is currently ongoing in order to promote anti-tumor immunity [108].

A recent in vitro study in chronic HBV infection revealed an immunosuppressive cascade in which HBsAg generated suppressive monocytes with higher levels of PD-L1 and HLA-E that can induce regulatory NK-cell differentiation leading to T-cell inhibition [110].

## 6. NK-T Cell Interplay in Chronic HBV Infection

In patients with chronic HBV infection, the overall NK cell effect in the control of the virus can be negatively affected by their regulatory activity on HBV-specific T cells.

In this regard, in vitro depletion of NK cells from PBMC of chronic patients improves HBV-specific (but not CMV-specific) T-cell responses, which indicates an active NK-cell suppression on HBV-specific T cells, which may contribute to the T-cell exhaustion typical of chronic HBV infection [48,105].

Interaction of up-regulated TRAIL ligand on NK cells and TRAIL-R2 on CD8 T cells, particularly within the liver, is directly involved in this suppressive activity [105]. Activated HBV-specific T cells upregulate TRAIL-R2 that makes them vulnerable to being killed by NK cells. A significant increase of CD8 T-cell reactivity is observed after the TRAIL pathway was blocked.

Another mechanism involved in the regulation of HBV-specific T cells by NK cells is mediated by NKG2D binding to its specific ligand on CD4 cells. HBV-specific CD4+ T cells upregulate the NKG2D ligand MICA, which leads to NK-cell activation and, consequently, to degranulation and cytotoxicity. In addition, an NKG2D blockade could rescue HBV-specific and MICA/B expressing T cells within the HBV-infected liver [102].

Nucleos(t)ide analogue therapy can improve T-cell responses and can modulate the inflammatory NK-cell phenotype of chronic HBV patients. NK depletion and blockade of TRAIL and NKG2D further ameliorate the HBV-specific T-cell functions [48]. Instead, IFN-stimulating agents, such as TLR7 agonists, can activate NK cells as well as cytotoxicity and cytokine production with a loss of the NK inhibitory effect on T cells [97]. In fact, the TLR7 agonist can efficiently enhance NK-cell activation in CHB patients treated with NUCs that lead to improved NK-cell antiviral potential, as shown by the increased cytokine production and degranulation capability, and a decreased NK-cell inhibitory effect on HBV-specific T cells. In this setting, NK cell activation may act synergistically with HBV-specific T cells in HBV control without detrimental effects for T-cell antiviral activity. The evidence that, in some groups of treated chronic patients, NK cells are weakly suppressive for HBV-specific T cells despite strong stimulation of NK cell activation, cytotoxicity, and cytokine production induced by therapy, which suggests that effector and suppressive NK cell functions are regulated by distinct mechanisms [97].

## 7. Final Remarks and Potential Clinical Applications

NK cell dysfunction has been described in both chronic infections and cancer models and may contribute to viral persistence and tumor growth [15,123]. Tumor-infiltrating NK cells usually exhibit an exhausted state and are prone to apoptosis, as a consequence of the immuno-suppressive effect of the tumor micro-environment [123]. In addition, NK cells can limit T-cell responses, by protecting the host from fatal immunopathology during viral infections. However, this can also diminish the antiviral activity of effector T cells, which favors the acquisition of an exhausted T-cell phenotype. Therefore, therapeutic approaches aimed at promoting the protective over the pathogenic and inhibitory effects of NK cells can represent a potential strategy to restore dysfunctional immune responses, by complementing T-cell immunotherapies. Different NK cell-based approaches are currently investigated in preclinical and clinical studies in the cancer setting with encouraging results. Among them, a blockade of immune checkpoints shared by T and NK cells represents a strategy with the potential to target both immune populations. For example, the PD-1/PD-L1 blockade has often been studied in combination with other regulatory receptor blockades, as the NKG2A/HLA-E inhibitory pathway, which is currently being tested in a clinical trial in patients with squamous cell carcinoma of the head and neck [108]. In addition, TIGIT, Tim-3, or the TGF-β blockade have been proposed to improve the effects of PD-1 inhibition in an NK cell-dependent manner in different mouse and human models of cancer [124,125]. The innate capacity of NK cells to preferentially target transformed cells translates into a reduced risk of autoimmune clinical events by using NK-related checkpoint inhibitors in the setting of hematological and solid cancers, compared to T cell-based therapies [125].

A different strategy was utilized to enhance the NK cell number and function, and is currently being investigated in clinical trials for cancer immunotherapy, which involves the in vivo administration of cytokines, i.e., IL-15, IL-12, and IL-18, that are known as NK-cell stimulatory factors. Cytokines have been tested alone or in association with adoptive transfer of autologous or allogeneic haplo-identical NK cells [126]. However, by using this approach, often in combination with other anti-tumor treatments, clinical efficacy is still limited due to the short lifespan of NK cells in vivo and the Tregs expansion caused by IL-2 administration [127,128]. Cytokine pre-activation has been demonstrated to induce an efficient development of memory-like NK cells, with an enhanced functionality in patients with acute myeloid leukemia. Importantly, expanded cytokine-induced memory-like NK cells displayed robust anti-tumor activity, which achieved a clinical response in more than half of the treated patients in the phase I clinical trial [129]. The potential immunotherapeutic benefits of memory-like NK cells transfer include long-term in vivo expansion and persistence and increased IFN-γ production and cytotoxicity against target cells. This makes them a promising NK cell-based modulatory intervention [130].

Moreover, a superagonist complex of IL-15 and IL-15α named ALT-803 has been used to activate NK cells and promote NK cell functions against hematologic tumors and solid cancers with good therapeutic efficacy [131,132].

Lastly, following the successful application of chimeric antigen receptors (CARs) to T cells, genetic engineering of CARs has also been applied to NK cells (CAR-NK). This allows them to recognize specific tumor-associated antigens with increased survival, proliferation, and cytotoxicity. So far, such a strategy is still in a pre-clinical phase, and numerous studies have been devoted to assess its clinical efficacy in cancer immune-therapy [133]. CAR-T cell clinical management still remains technically difficult to apply to large numbers of patients due to their complex preparation, costs, and safety limitation. CAR-NK cells, instead, have several advantages that allow them to overcome these problems. Notably, engineered CAR-NK cells preserve their activating and inhibitory receptors, and decrease the risk of relapses following the downregulation of the CAR-targeting antigen. In addition, allogenic NK-cell infusion lacks the potential to induce graft-versus-host (GVHD) reactions, which allows the use of a broader spectrum of cell sources for NK-cell immunotherapy [133].

While the therapeutic manipulation of NK cells has already reached the clinics for several tumors, the potential positive contribution of NK-cell modulation in the setting of persistent HBV infection needs to be further explored.

In HBV infection modulation of the NK-cell function is expected to be useful not only to correct NK-cell dysfunction and improve NK cell anti-viral activity but also to abrogate the negative NK-cell effect on T cells, which is believed to contribute to T-cell exhaustion [48,105]. The analysis of the immune modulatory effect of the TLR-7 agonist therapy on T-cell and NK-cell responses of HBeAg-virally suppressed chronic HBV patients supports this possibility and provides evidence that positive and detrimental NK-cell functions can be dissected by therapy. TLR7 agonist therapy was able to potently activate NK-cell cytotoxicity and cytokine production, which have the potential to be protective [97], but reduced NK-cell inhibitory activity on HBV-specific T cells, which was easily detectable before starting therapy, but significantly declined during TLR7 agonist treatment [97]. Despite the efficient stimulation of the NK-cell antiviral function and the abrogation of the NK-cell inhibitory effect, TLR7 agonist therapy failed to induce HBsAg seroconversion, which suggests that this immune modulatory approach may not be sufficient alone to significantly affect host protective responses and allow viral control.

Additional studies are, thus, needed to unravel more deeply the complex T cell/NK cell interplay in the perspective of developing specific strategies to selectively modulate the different NK-cell functions. What our present understanding of T and NK cell biology suggests is that the final net result of positive and detrimental NK-cell activities depends not only upon the power of the NK suppressive effect but also on the level of protection that T cells can afford. The study of different clinical conditions where the balance between “good and bad” NK cell activities may be different (as, for example, in TLR-treated patients) is certainly key to define the mechanisms that drive the activation of different NK-cell functions and understand whether NK-cell modulation can be of real benefit for an HBV cure.

In this context, NK-cell modulation could be combined with drugs more selectively active on adaptive responses, such as specific vaccines, or on HBV replication directly, in the perspective of therapeutic strategies based on the association of different compounds able to express complementary anti-viral activities [3,134,135].

## Figures and Tables

**Table 1:** Summary of described NK cell subsets. The upper four rows report NK cell subsets are identified among circulating NK cells. Rows filled in grey depict NK subsets recognized within the liver.

NK Cell Subset	Phenotypic Characteristics	Functional Activity	Comments
CD56^bright^ NK circulating cells	CD56^bright^ CD16^neg/low^	High cytokine production Low cytolytic activity	Considered as precursors of the more mature CD56 ^dim^ NK cells [13]
CD56^dim^ NK circulating cells	CD56^dim^ CD16^bright^	Cytolytic activity Low cytokine production	Main circulating NK subset Terminally differentiated NK cells [13]
	CD56^dim^ CD16^neg^	High cytolytic activity High cytokine production in healthy individuals	Not fully functional in malignancies [17,18]
CD56^neg^ NK circulating cells	CD56^neg^ CD16^bright^	Low cytolytic activity Low cytokine production	Minor subset in healthy donors Significantly expanded in chronic HIV and HCV chronic infections [19] and in CMV/EBV co-infected older healthy donors [20]
Hepatic conventional NK cells	CD56^dim^ CD16^bright^ CCR5^neg^ CXCR6^neg^	High cytolytic activity [21]	Similar to peripheral CD56^dim^ [22] Promote T cell function [21]
Liver-resident NK cells	CD56^bright^ CD16^low^ CD69^+^ Tbet^low^ Eomes^hi^CCR5^+^ CXCR6^+^	low levels of perforin and granzyme B Low cytokine production Low cytolytic activity [23]	Regulatory role through the PD1/PD-L1 signaling [21] Can acquire memory to haptens and viral antigens [24,25]

**Table 2:** Mechanisms of NK/T cell interplay. Indirect and direct mechanisms of NK/T-cell interaction are summarized and divided based on the resulting T-cell response enhancement or inhibition. References relative to human or animal studies are reported.

Mechanisms of NK/T Cell Interplay			Animal Studies	Human Studies	HBV Studies (human)
Indirect mechanisms	enhancement	DC maturation and IL-12 production		[77,82,83,84]	
		DC recruitment	[87]	[86]	
		Promoting Ag cross-presentation by DC	[88]	[89,90]	
	inhibition	APC capacity reduction	[91]		
		DC killing	[92,93]	[94]	
		Ag availability modulation	[95]		
Direct mechanisms	enhancement	***a.****Cytokine-mediated interaction*** Anti-viral/pro-inflammatory cytokine secretion	[96]	[96]	[97]
		***b.****Receptor/Ligand NK-T cell cross-talk*** T cell protection by:			
		2B4/CD48	[98,99]		
		NKG2A/HLA-E or Qa-1b	[100]		
	inhibition	***a.****Cytokine-mediated interaction*** IL-10/TGF-β secretion	[79]	[79]	
		***b.****Receptor/Ligand NK-T cell cross-talk*** T cell killing by:			
		NKG2D/NKG2DL	[80,81]	[101]	[102]
		DNAM-1/PVR		[103]	
		TRAIL/TRAIL-R2	[104]		[48,105]
		NCR1/NCR1-L	[106,107]		
		***c.****Checkpoint inhibitory pathways***			
		PD-1/PD-L1	[108]	[108]	
		NKG2A/HLA-E or Qa-1b		[109,110]	
